# Supplementary material for: Active site specificity profiling datasets of matrix metalloproteinases (MMPs) 1, 2, 3, 7, 8, 9, 12, 13 and 14
Source: Data Brief. 2016 Feb 22;7:299–310. doi: 10.1016/j.dib.2016.02.036 (PMC4777984; doi:10.1016/j.dib.2016.02.036)
Supplement: Supplementary file 10 — Supplementary material [file mmc10.zip › WebPICS_hMMP14_T_1%/P3.html]

 

PICS results


|  |  |
| --- | --- |
| **P3\_A**  31 in 307 sites   10.1 %    effects > 10 perc. pnts.  (vice-versa in brackets)  P2\_N: 15.8 (23.2) |  |
| **P3\_H**  8 in 307 sites   2.6 %    effects > 10 perc. pnts.  (vice-versa in brackets)  P1\_D: 31.0 (12.4)   P2prime\_M: 20.1 (10.7) |  |
| **P3\_I**  21 in 307 sites   6.8 %    effects > 10 perc. pnts.  (vice-versa in brackets)  P2\_Q: 17.0 (17.0)   P2\_S: 16.3 (14.9) |  |
| **P3\_L**  30 in 307 sites   9.8 %    effects > 10 perc. pnts.  (vice-versa in brackets)  P2prime\_I: 15.3 (13.1) |  |
| **P3\_S**  21 in 307 sites   6.8 %    effects > 10 perc. pnts.  (vice-versa in brackets)  P1prime\_Q: 14.4 (21.8)   P2\_E: 16.3 (14.9)   P3prime\_V: 15.3 (12.4) |  |
| **P3\_V**  22 in 307 sites   7.2 %    effects > 10 perc. pnts.  (vice-versa in brackets)  P1prime\_M: 12.7 (16.3) |  |
